# Transport of airborne *Picea schrenkiana* pollen on the northern slope of Tianshan Mountains (Xinjiang, China) and its implication for paleoenvironmental reconstruction

**DOI:** 10.1007/s10453-012-9270-2

**Published:** 2012-08-01

**Authors:** Yanfang Pan, Shun Yan, Hermann Behling, Guijin Mu

**Affiliations:** 1 Xinjiang Institute of Ecology and Geography, Chinese Academy of Sciences, Beijing Road 818, Urumqi, 830011 China; 2 Department of Palynology and Climate Dynamics, Albrecht-von-Haller-Institute for Plant Sciences, University of Göttingen, Untere Karspüle 2, 37073 Göttingen, Germany; 3 Qira National Field Research Station for Desert Steppe Ecosystems, Chinese Academy of Sciences, Qira County , 848300 Hotan, Xinjiang China

**Keywords:** Cour method, Pollen dispersion, Sandstorm, Aerobiology, Climate change, Vegetation history, Arid regions

## Abstract

The understanding of airborne pollen transportation is crucial for the reconstruction of the paleoenvironment. Under favorable conditions, a considerable amount of long-distance-transported pollen can be deposited far from its place of origin. In extreme arid regions, in most cases, such situations occur and increase the difficulty to interpret fossil pollen records. In this study, three sets of Cour airborne pollen trap were installed on the northern slope of Tianshan Mountains to collect airborne *Picea schrenkiana* (spruce) pollen grains from July 2001 to July 2006. The results indicate that *Picea* pollen disperses extensively and transports widely in the lower atmosphere far away from spruce forest. The airborne *Picea* pollen dispersal period is mainly concentrated between mid-May and July. In desert area, weekly *Picea* pollen began to increase and peaked suddenly in concentration. Also, annual pollen indices do not decline even when the distance increased was probably related to the strong wind may pick up the deposited pollen grains from the topsoil into the air stream, leading to an increase of pollen concentration in the air that is irrelevant to the normal and natural course of pollen transport and deposition. This, in turn, may lead to erroneous interpretations of the pollen data in the arid region. This study provided insight into the shift in the *Picea* pollen season regarding climate change in arid areas. It is recorded that the pollen pollination period starts earlier and the duration became longer. The results also showed that the temperature of May and June was positively correlated with the *Picea* pollen production. Furthermore, the transport of airborne *Picea* pollen data is useful for interpreting fossil pollen records from extreme arid regions.

## Introduction

The genus *Picea* is one of the dominant tree species in boreal and cold-temperate evergreen coniferous forests, consisting of 35 species. The genus is broadly distributed in subalpine vegetation of temperate, cold-temperature, and subtropical climate zones in the Northern Hemisphere. Taxa occurring in China include 16 species and 9 varieties. At present, spruce (*Picea*) as a cold-adapted tree only grows in a few high mountains in North China. The history of spruce forests can be a valuable tool for understanding environmental changes, ecological restoration, and human impacts on the environment because of its sensitivity to climatic variability, paleo-drought, and human-induced deforestation.

Pollen, foliage, leaves, and cones of *Picea* have been found in the late Cenozoic, which indicated that *Picea* was present as flora during Pliocene to early Pleistocene and widely distributed in the subalpine of Tibetan Plateau (Kong et al. 1996; Lu et al. 2001). Moreover, *Picea* pollen appeared in samples of the low mountain area of eastern, central, western, and southern China, even on plains during the Quaternary period, especially during the last glaciation. By using the change of spatial and temporal distribution characteristics of *Picea*, paleoclimate, paleovegetation, and paleoenvironment have been reconstructed in China (Li and Zhou 1979; Wang 1990; Wang and Wu 2000; Liu et al. 2002). However, the pollen–vegetation relationships are dependent on a number of factors, of which the most important are the pollen production of individual species, pollen dispersal, the spatial distribution of vegetation around the sampling site, and the basin size of the sampling site.

*Picea* pollen grains are anemophilous with characteristic air-filled sacs that support their dispersal (Niklas 1985), but compared with *Pinus*, Jonassen (1950) indicates that *Picea* is poor in pollen production and hence ineffective in dispersal and colonization. Because of large and heavy *Picea* pollen grains, they are not as easily dispersed by wind as, for example, *Pinus* pollen, despite the availability of their air sacs (Erdtman 1969; Hicks 2001, Brostrom 2002). Furthermore, according to Eisenhut (1961), the fall speed of *Picea* pollen grains (c. 6 cm/s) is almost twice as high as *Pinus* (3.1–4.5 cm/s). Therefore, it may be assumed that a large proportion of *Picea* pollen will be deposited within less than 1 km of the parent tree.

The Xinjiang region, situated in the hinterland of Eurasia Continent, is the largest and driest area in China. The uplift of the Tibetan Plateau holding up the warm, humid airflow from the Indian Ocean, which leads to precipitation in this region that is much less than other regions of China. Xinjiang region spans over 1.6 million km^2^, but the forested area accounts only for 2.94 %. About 85 % of the tree species in the area are coniferous forest types, and *Picea* is the main species in this coniferous forest. *Picea* species occurring in Xinjiang region are only *Picea schrenkiana* and *Picea obovata*. The former is mainly distributed in the northern and southern slope of the Tianshan Mountains and rarely in the west of the Kunlun Mountains, the latter mainly distributed in southwest slope of the Altai Mountains. *Picea* species are not abundant and their distribution is limited in Xinjiang area (Li 1991).

**Fig. 1:**
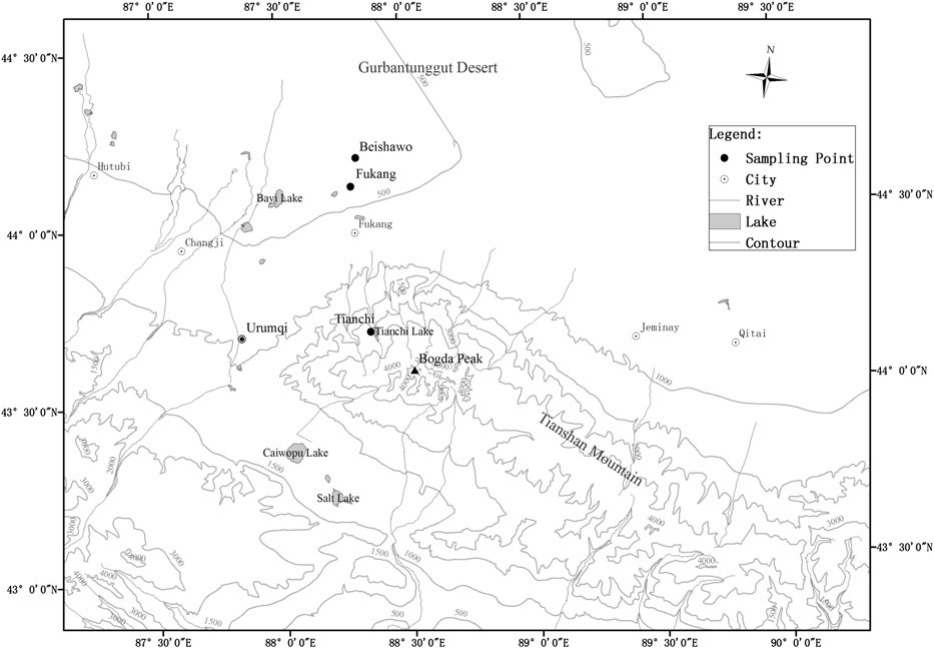
Location of the three sampling sites

On the other hand, the specific topography provides us with a good place to study the transport of the *Picea* pollen (Fig. 1). The topography of Xinjiang can be summarized as two basins clipped in three mountains with the Altai Mountains in the northern part, the Kunlun Mountains in the southern part, and the Tianshan Mountains lying across the central. Junggar Basin is clipped between the Altai Mountains and the Tianshan Mountains, and the Gurbantunggut Desert in the middle of the basin; in the middle of the Kunlun Mountains and the Tianshan Mountains lies the Tarim Basin, and the Taklimakan Desert is in the middle of the basin.

The reconstruction of vegetation dynamics in the past needs the knowledge of modern pollen and vegetation relationship as accurate as possible. Modern *Picea* pollen studies, which have been conducted in Xinjiang, were based on surface soil collection. *Picea* pollen appeared in the surface soil samples of all the vegetation zones in Xinjiang (Yan et al. 2004; Li 1991), and distance is the most important factor that affects the content of *Picea* pollen in surface soils in this region. *Picea* pollen can be scattered beyond 700 km away from the spruce forest stand. Especially, in desert and desert-steppe, the *Picea* pollen content is relatively higher. This indicates that *Picea* pollen disperses extensively and is transported widely. But because of its origin, transportation and deposition of *Picea* pollen are still unclear. Such descriptive or quasi-quantitative studies could not reflect the quantitative relationships between pollen surface samples and vegetation, especially in regions around the Tianshan Mountains, where various vegetation types and a unique topography existed. However, there are many differences between paleovegetation and paleoenvironments, which were reconstructed using fossil and surface soil pollen. With the intention of interpreting the fossil record as objectively as possible, modern pollen influx data are used for the calibration of influx diagrams. Contemporary *Picea* airborne pollen data can help to identify both source areas of pollen transported over long distances and the dominant wind direction that can help the reconstruction of the vegetation history (Gassmann and Pérez 2006; Rousseau et al. 2006). Moreover, airborne pollen data can also be used to monitor the phenology of the vegetation. Such information can then be applied to identify and interpret possible climatic and environmental changes (Molina and Palacios 2001; Bortenschlager and Bortenschlager 2005; Damialis et al. 2007) on a local as well as on a regional scale. Until now, no study on the relationship between airborne *Picea* pollen and climate in China exists.

Three Cour airborne pollen traps (Cour 1974) located at Tianchi Weather Station, Fukang Research Station of the Chinese Academy of Sciences, and Beishawo for Multisphere Observation and Research Station were used to collect airborne pollen in the area provided samples (from July 2001 to July 2006), which analysis was focused on *Picea* pollen. The aim was to quantify the airborne pollen grains and establish the pollen season to measure the pollen dispersion and to study the relationship between meteorological parameters in the northern slope of Tianshan Mountains. The final goal is to use the results to improve the reliability of modern pollen–climate transfer functions and provide a more robust baseline for analysis of fossil *Picea* pollen assemblages.

## Study areas

**Fig. 2:**
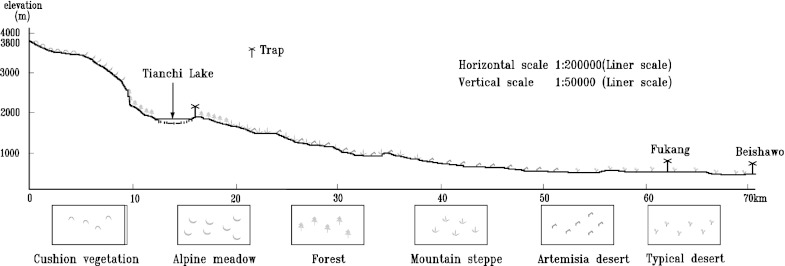
Trap site and vertical distribution of vegetation on the northern slope of Tianshan Mountains

The study area is situated in the northern slopes of Tianshan Mountains in Xinjiang and displays a clear vertical distribution of vegetation types (Figs. 1 and 2). The vertical zones can be described as follow: From the higher to the lower elevated areas, vegetation includes alpine cushion, alpine and subalpine meadow, Tianshan spruce forest, steppe and desert-steppe, semidesert (*Artemisia* desert), and typical desert zones (Team of Investigation of Xinjiang 1978; Editorial Committee of Xinjiang Forest 1989). A forest vegetation zone is located from 1,720 to 2,700 m a.s.l.. Generally, forest, consisting mostly of *P. schrenkiana*, accompanied by *Aegopodium alpestre* and *Calamagrostis arundinacea* is restricted to moist areas on the north-facing slope in the zone (Yan 2002).

The Tianchi weather station is at 43°53′N, 88°07′E, 1,942.5 m a.s.l.. The climate is characterized by an annual mean temperature of (1977–2006 average) −11 °C in January and 15 °C in July. The mean annual temperature is 2.04 °C. The mean annual rainfall is approximately 550 mm (1977–2006 average). The mean annual non-frost period is 88.6 days, and the mean relative humidity is 56–64 %. The forest of the *P. schrenkiana* population in our study is distributed from 1,500 to 2,700 m a.s.l. on the center of Tianshan Mountain (Zhang and Tang 1989). Some broadleaf trees and shrubs, for example, *Sorbus tianschanica*, *Salix xerophila*, *Betula tianschanica*, *B.* *verrucosa*, and *B. microphylla*, are also found in the forest.

The Fukang Station of Desert Ecology (44°17′N, 87°56′E, 477 m a.s.l.) is located at south fringe of Zhungaer Basin, at the foot of highest peak of eastern Tianshan Mountains, the Bogeda Peak. The station is at a 55 km distance from Tianchi station. The climate of the plain area is typical continental arid, with hot–dry summer and cold winter. The highest recorded temperature is 46 °C while the lowest is −41.6 °C. Annual precipitation is around 150 mm and annual pan evaporation is around 1,000 mm. Frost-free days per year are about 170.

The Beishawo experimental site (44°22′N, 87°56′E, 443 m a.s.l) is at the southern edge of the Gurbantunggut Desert. The mean annual precipitation is no more than 150 mm, falling predominantly during spring. Mean annual evaporation is more than 2,000 mm. Average temperature is 6.6 °C and extreme temperature is higher than 40 °C. Small half-arbors composed of *Halaxylon persicum, H. ammodendron*, and other desert plants are developed widely.

## Materials and methods

Airborne *Picea* pollen was collected using three sets of Cour pollen trap (Cour 1974) installed at Tianchi Weather Station, at a height of 1.5 m above ground, at the Fukang Research Station of the Chinese Academy of Sciences and Beishawo Field Station. The trap monitored the air of the station uninterruptedly for five consecutive years (from July 2001 to July 2006). The Cour trap uses 400 cm^2^ sterile gauze filters impregnated with silicone oil and fitted to a vane. The apparatus includes an anemometer indicating the wind flow through the filter during the exposure period. The set of filters was exposed all year long. Filters were changed once a week from May to October, and once every 2 weeks from November to April of the following year. Sample processing was done according to the methodology proposed by Cour (1974): the filters are subjected to a series of chemical treatments in the laboratory to destroy the gauze and release the pollen, which is then acetolysed and colored to help in its identification. The chemical treatment, simplified, is as follows: cold sulfuric acid, cold hydrofluoric acid (70 %), hot hydrochloric acid, hot potassium hydroxide, acetolysis by the Erdtman method (Erdtman 1960) (sulfuric acid and acetic anhydride at about 100 °C for 3 min), and coloration with basic fuscin. The microscopic identification of *Picea* pollen was performed according to the pollen types described by Wang et al. (1995) under a magnification of 250. Pollen counts were made once a week or once every 2 weeks. The total weekly counts were converted to the number of pollen grains per cm^2^. The monthly total grain was the sum of all weekly pollen grain per month, while the total annual pollen grain was the sum of all monthly counted grains per year. When accounted the *Picea* pollen season, the daily pollen data are averaged over 7 days (Alix 2002). In this paper, according to the method described by Nilsson and Persson (1981), the main pollen season was defined as the period from the day when the daily sum of *Picea* pollen concentrations reaches 5 % of the total annual *Picea* pollen sum until the day when the daily sum reaches 95 % of the total annual *Picea* pollen sum.

At the same time, weather stations were mounted on the stations to record meteorological data, including temperature, precipitation, wind direction, and velocity.

## Results and discussion

### Weekly and monthly airborne *Picea schrenkiana* pollen dispersal

**Fig. 3:**
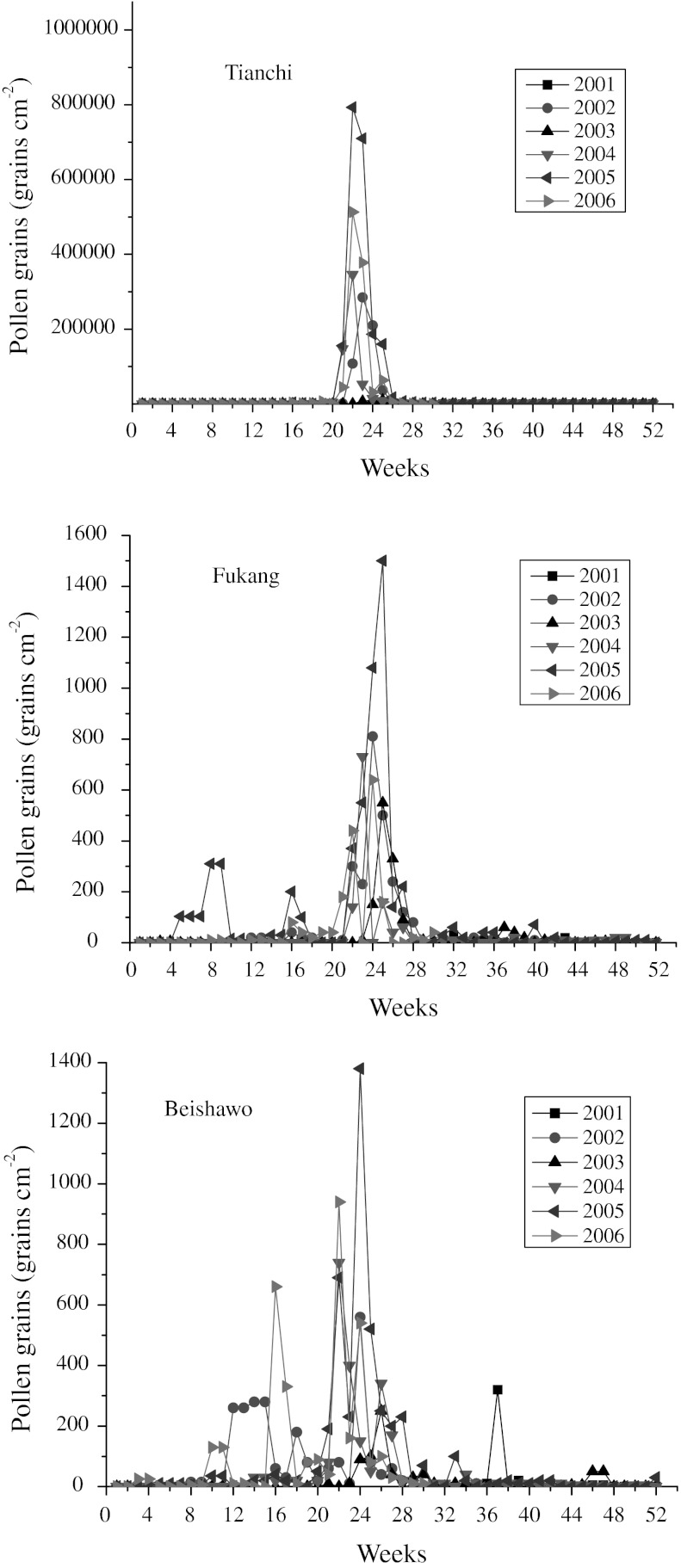
Weekly *Picea* pollen counts in Tianchi, Fukang, and Beishawo stations 2001–2006

**Fig. 4:**
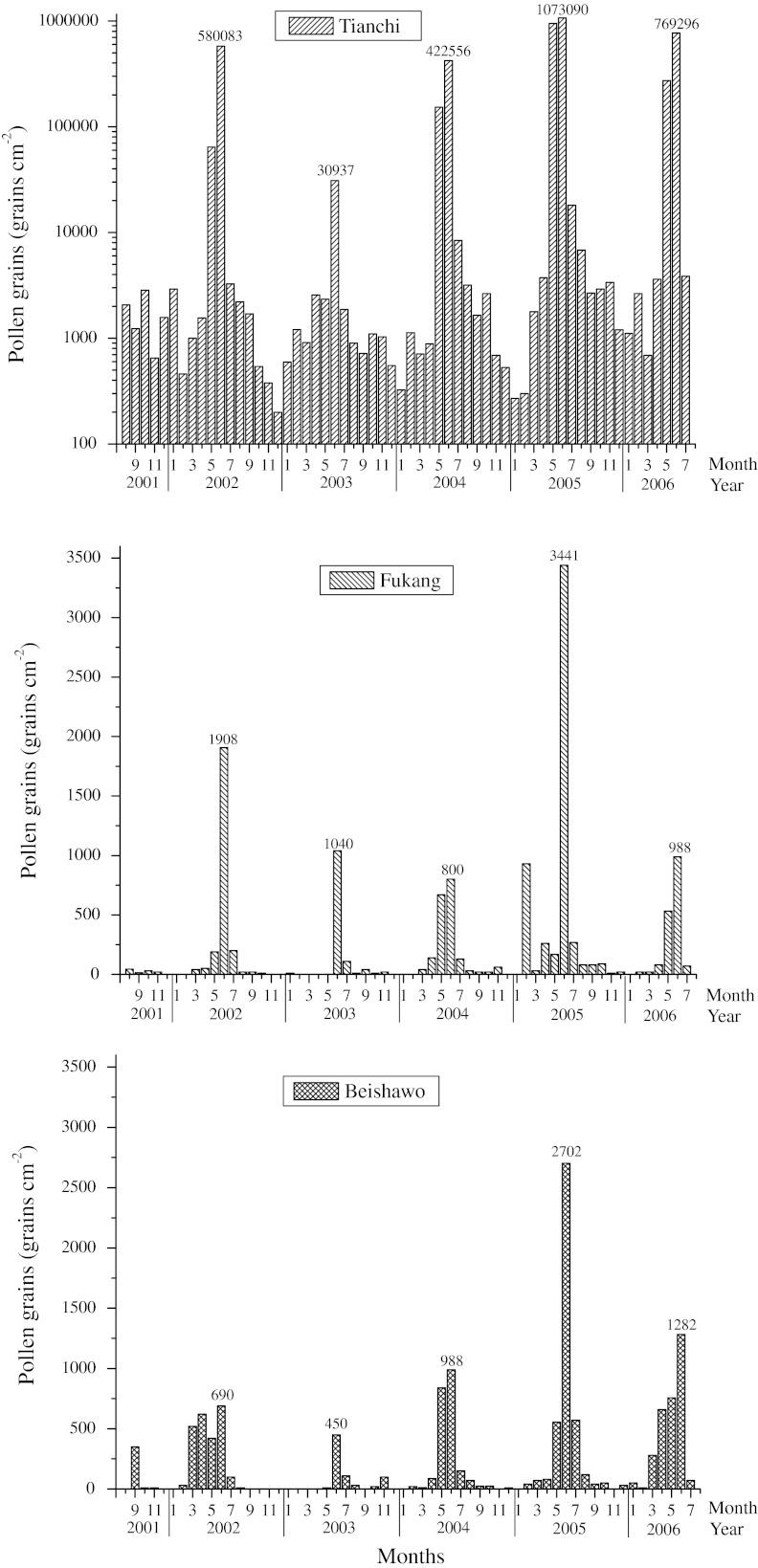
Monthly *Picea* pollen counts in Tianchi, Fukang, and Beishawo stations 2001–2006

Figures 3 and 4 show the characterization of the weekly and monthly airborne *Picea* pollen grains measured in the air above Tianchi, Fukang, and Beishawo station during the study period. According to the comparison between Tianchi and Fukang, and Beishawo, *Picea* pollen grains exist in the atmosphere of Tianchi year-round, the counts ranged from 20 to 793,320 per cm^2^. The top value for a pollen peak was recorded during 22 weeks in 2005 in Tianchi (793,320 grains, Fig. 3; Table 1). Another important difference is that in May and June 2003, the weekly counts were very low compared with the corresponding values for the same week/month in other years. Generally, the curve in Tianchi is characterized by a sharp increase in pollen grains, maximum values for about 1 month and a rapid decrease, and more than 90 % of the pollen grains were collected during this period in Tianchi station. The pollen decreased gradually thereafter, reaching its lowest level in December and January, and then increased slowly starting in February.

**Table 1:** Position of the week with the maximum *Picea* pollen count (week max = week of the peak)

Years	2001–2002	2002–2003	2003–2004	2004–2005	2005–2006
Tianchi	23 (28,200)	25 (10,950)	22 (346,080)	22 (793,320)	22 (513,320)
Fukang	24 (810)	25 (550)	23 (730)	25 (1,080)	24 (640)
Beishawo	24 (560)	26 (250)	22 (740)	24 (1,380)	22 (940)

For the Fukang station, the pollen grains ranged from 0 to 1,500 *Picea* pollen grains per cm^2^, and for Beishawo, from 0 to 1,380 *Picea* pollen grains per cm^2^ (Fig. 3; Table 1). During the years studied, the highest pollen counts were always recorded from May to July (Fig. 4), a period in which 69–86 % in Fukang and 40–81 % in Beishawo of the total annual pollen catch was collected.

As the year progressed, one *Picea* pollen peak occurred in Tianchi, while several other pollen peaks were detected in Fukang and Beishawo (Fig. 3). The highest, which usually occurred during June (in the *Picea* pollination period), could be correlated to long-distance transport of airborne *Picea* pollen from the Tianshan mountains’ spruce forest. Small peak *Picea* counts were always detected in February to May, so we speculate that pollen grains were transported from redeposition.

### The position of the *Picea schrenkiana* pollen peak

The position of the pollen peak is the week where the maximum value of pollen grains in the air is recoded. It is a very important set of data for *Picea* pollen dispersal and long-distance transport. During the years 2001–2006 (Table 1), the week in which the highest quantities of pollen were recorded in Tianchi station was as follows: In 2002, *Picea* pollen counts peaked at week 23, while for 2004, 2005, and 2006, the highest score was detected during week 22. Furthermore, the peak counts are usually recorded only a week after the start of the pollen season. In this context, 2003 appears to have exceptions (the pollen peak occurred later, during week 25), which may be explained by the low temperature affecting pollination. However, the pollen peaks in Fukang and Beishawo were varied year to year; the maximum value occurred during weeks 22 and 26, generally later than in Tianchi.

Because of the assumed hypothesis about the origin of the transported *Picea* pollen, the maximum wind speed and direction of wind were taken into consideration. The prevailing winds were northwest wind in Fukang and Beishawo station. Through the Pearson Correlation analysis in Beishawo, the week the maximum value of pollen grains was negatively related to the maximum wind (correlation = −0.084). In each year, the angle at which the maximum weeks arrived from was noted, and although this angle varied, in general, it corresponded to an N–NW direction that hinders the airborne *Picea* pollen transported from Tianshan spruce forest to the desert region. Only in 2003 and 2006 were different, corresponding to S–SW directions in Beishawo station, the pollen counts peaked at week 22.

There are about 55 km between Tianchi and Fukang, and 70 km of Beishawo, but we find that the highest quantities of pollen did not decline even as the distance increased, as the dispersal of airborne pollen grains depend both on the weather conditions and on the micro and macro topography.

### Seasonality of total airborne *Picea schrenkiana* pollen dispersal

The seasonal variation of pollen is mainly controlled by the phenology. The data from the three stations (Fig. 4) indicated a regular variation in the *Picea* pollen content according to seasons, with the highest average counts for each year during the summer (June through August) and the spring (March through May), while the lowest average grains was found during the winter (December through February).

Table 2 shows the abundance of *Picea* pollen in Tianchi, Fukang, and Beishawo stations. The data show the highest annual pollen influx in Tianchi, which is related to the presence of spruce forest. The highest annual grains of *Picea* pollen was measured in 2004–2005, a total number of 2,055,860 grains per cm^2^ in Tianchi and the lowest in 2002–2003. *Picea* pollen showed an annual average of 885,228 grains in Tianchi, 2,558 in Fukang, and 2,560 grains in Beishawo. Comparing with influx of pollen grains in Tianchi, influx was low in Fukang and Beishawo, the amounts of airborne *Picea* pollen in Fukang and Beishawo were less than 5 % of the amounts in Tianchi (Table 2). The annual average percentages of *Picea* pollen rose up to 28.85 %, an average of 21.15 % in Tianchi, and the annual average percentage of *Picea* pollen in Fukang and Beishawo were less than 3 %, even in May and June, they did not increase to no more than 5 % (Mao 2008). The annual airborne of *Picea* pollen grains changed greatly between mountains and lowlands indicating that, a large proportion of *Picea* pollen will be deposited within the parent tree and wind has a very limited capacity to carry a large number of *Picea* pollen grains from mountains to the lowlands. It is confirmed that, *Picea* species produce heavy pollen grains with a relatively high fall speed and hence long-distance transport of pollen is limited (Sugita et al. 1999). Based on our airborne pollen data, we are convinced that, when interpreting paleoenvironments, a higher percentage of *Picea* seems to correspond to a larger extent of coniferous forest in the mountains and a denser vegetation cover in the lowlands (Zhu et al. 2001).

**Table 2:** The total annual sums of airborne *Picea* pollen observed during study period

Station	2001–2002	2002–2003	2003–2004	2004–2005	2005–2006	Mean
Tianchi	662,080	45,460	591,210	2,055,860	1,071,530	885,228
Fukang	2,500	1,210	1,860	5,230	1,990	2,558
Beishawo	2,750	580	2,250	4,150	3,350	2,560

### *Picea schrenkiana* pollen transport and its implication for paleoenvironmental reconstruction

Pollen dissemination in entomophilous taxa is restricted to the range of pollinators. While anemophilous pollen is often carried by great distances, sometimes tens of kilometers, before setting out of the atmosphere (Erdtman 1937). *Picea* pollen can be transported over long distances. Fukang and Beishawo airborne *Picea* pollen data (Table 2) showed wind can transfer pollen from source population of *Picea* forest over at least 70 km. This indicated that *Picea* pollen disperses extensively and transports widely. But distance also affects *Picea* pollen dispersal in the air. Compared with pollen data from the Tianchi station, there is little pollen in Fukang and Beishawo station in the *Picea* pollination period. The numbers of pollen grains decline with increasing distance from the source (Figs. 3, 4). *Picea* pollen is transported over much larger distances and is observed in lower quantities at a considerable distance from the source. This is coinciding with studies of surface sample from the Nei Mong Gol, Gansu, and Xinjiang regions (Yan et al. 2004; Li 1991; Zhu et al. 2001).

Long-distance pollen transport can also influence the total yearly pollen count and seasonal duration. The analysis of annual *Picea* pollen grains in the three stations illustrates that *Picea* pollen production is one of the most important factors affecting airborne pollen dispersal. The pollen indices in Fukang and Beishawo stations varied following the variation in Tianchi: the more *Picea p*ollen grains in Tianchi station, the more in Fukang and Beishawo station. We also found that, annual *Picea* pollen grains did not decline even as distance increased from *Picea* forest. There are about 55 km between Tianchi and Fukang, and 70 km of Beishawo, but we found that annual *Picea* pollen grains did not decline even as the distance increased, even for several years (2003–2004 and 2005–2006). The total *Picea* pollen accounts in Beishawo were higher than in Fukang. The mean account of 5 years (2001–2006) airborne pollen collection in Beishawo was more than in the Fukang station. The deposited particles can be resuspended or refloated into the atmosphere following turbulent climatic events, such as strong wind. In central Hokkaido, Japan, refloated *Pinus* pollen comprised 0.5–22 % of the total arboreal count (Igarashi 1987). In this study, refloating of *Picea* pollen grains occurred beyond the *Picea* season in a large account (14–31 % in Fukang and 19–60 % in Beishawo of the total annual pollen was collected). This indicated that pollen atmospheric movement is a very complex phenomenon influenced by numerous environmental parameters. Wind velocity and direction as well as the topographic conditions are also important factors affecting dispersal efficiency of *Picea* pollen. However, due to incomplete meteorological data, wind impact on airborne *Picea* pollen transport has not been thoroughly studied.

Therefore, large amounts of *Picea* pollen grains are not usually transported very far from source areas. In spruce forests, the modern pollen assemblage is dominated by *Picea* pollen, but the percentage of *Picea* decreases sharply by distance from the forest and is usually lower than 2 % in most surface samples from shrublands, meadows, and grasslands (Li 1999; Shang et al. 2009). If the value of *Picea* pollen is greater than 15 %, spruce forest is usually inferred to exist nearby (Li 1998). However, *Picea* pollen can also be overrepresented, as the values of *Picea* pollen can be as high as 15 % in desert and tundra regions with little to no vegetative cover, even if the closest spruce forest is 10 km away (Cheng et al. 2004; Yang et al. 2004). Pollen production and concentration are very low in regions with little vegetation. Thus, *Picea* pollen can reach a relatively high proportion even though the absolute number of *Picea* pollen grains transported over such a long distance is small. More caution is needed in the procedure of paleovegetation reconstructions and environmental explanations based on pollen diagrams from fossil sample, especially in desert and desert-steppe. The content of *Picea* pollen in surface soils can not be said to represent the “real” vegetation in the area inferred from the pollen diagram.

Most pollen grains are deposited on the ground within a short distance of their source (Raynor et al. 1966; Gregory 1973). Between anthesis and the final deposition of pollen grains, however, several disturbances can occur which cause devotions from the normal and natural course of pollen deposition. This, in turn, may lead to errors in the interpretation of pollen data. In this study, *Picea* pollen grains began to increase and peaked suddenly in concentration in Fukang, especially in Beishawo, in March and April (Fig. 3). We speculated here that, in the desert region, strong wind may pick up the deposited pollen grains from the topsoil into the air stream, leading to an increase of pollen concentration in the air that is irrelevant to the normal and natural course of pollen transport and deposition. Fukang and Beishawo stations are located at the southern edge of the Gurbantunggut Desert where sandstorms occur frequently. Although the strongest wind usually occurs in spring, sandstorms occur during April to August, mostly in April and May (Wang et al. 2002). This is coinciding with the observations made by Ho et al. (2005). They found significantly higher total fungal spores associated with dust events in Hualien City. Similarly, turbulent weather contributed to evidence for long-distance dispersal of bacteria in Sweden, where exotic Bacillus species were isolated from red-pigmented snow (Bovallius et al. 1978). Back-trajectory analysis of wind and analysis of associated clay, fungal, and pollen particulates indicated an origin near the Black Sea, 1,800 km distant, where a sandstorm had occurred 36 h earlier.

Xinjiang is an area where sandstorms occur frequently. Dust from the Taklamakan and Gobi deserts (Xinjiang) is blown eastwards toward Korea, Japan, and the Pacific Ocean, affecting the Arctic, Hawaiian Islands, and the western coast of the USA (Duce et al. 1980). It is believed that such dust affects the pollen types and quantity in Japan. However, there have been no such studies on the relationship between the pollen dispersal and transport and the sandstorm conditions in Xinjiang.

In previous researches, Chenopodiaceae, *Artemsia, Ehedra*, and some other desert pollen types are found overrepresented in the Quaternary sediments and even the late Tertiary deposits in this region. This is often attributed to plant abundance and high productivity. However, based on airborne *Picea* data, we suggest that it is necessary for further study on the mechanisms of pollen transportation in Xinjiang. More pollen traps are needed to enlarge the current database and to tract the exact pathways of the pollen transport.

### *Picea schrenkiana* pollen season responses to climate change

Table 3 shows seasonal distribution of *Picea* pollen in Tianchi, the start of the main *Picea* pollen season over a five-year period occurred during the interval May 22–29. The end of the season was most variable ranging from June 18 (2004) to June 25 (2006). The duration of each season varied from 21 days to 211 between 2001 and 2006. In 2002 to 2003, the pollen season (Table 3) started on March 16 and stopped on October 12, with marked differences, in this case, compared with the 4 others years. During the 5 years, the start of the pollen seasons became earlier by about 7 days in Tianchi, the start date indicated a tendency toward earlier onset, although it is not statistically significant. The trend for pollen season end date also indicated a progressive delay. Consequently, pollen season duration tended to be longer.

**Table 3:** Seasonal distribution of *Picea* pollen in Tianchi, 2001–2006

	2001–2002	2002–2003	2003–2004	2004–2005	2005–2006
*Picea*					
5 %	29/5	16/3	25/5	23/5	22/5
95 %	19/6	12/10	18/6	23/6	25/6
Season duration	21	211	24	30	33

The seasonal dates where the accumulated sum since the beginning of the year reaches 5 and 95 %, and seasonal duration is shown in each case

Using the method, definition of pollen period by Nilsson and Persson, start date and end date of the pollen season, depends too much on the yearly pollen count. In another year, the total pollen grains in May and June reached more than 90 % of the whole year’s sum and the start date of *Picea* pollen season is in May. But for year 2002–2003, the total pollen grains in May and June account for only 73 %. Because the yearly pollen count changes every year, it is difficult to compare the start date of pollen seasons from year to year.

**Fig. 5:**
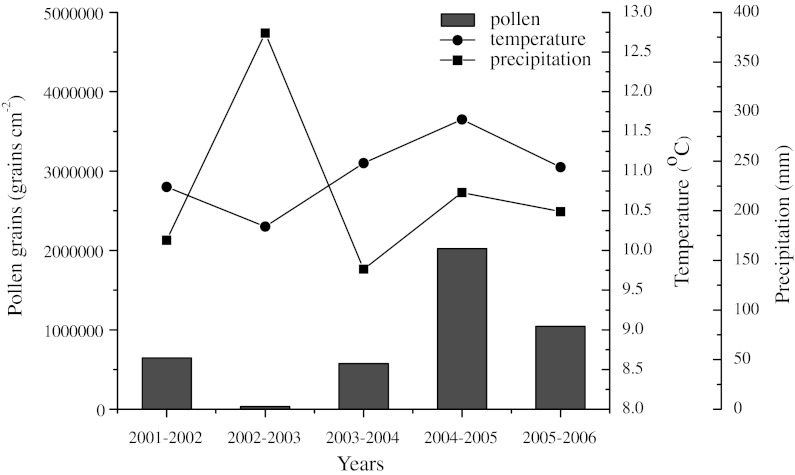
Monthly *Picea* pollen count with monthly average temperature and precipitation during May to June in Tianchi station in 2001–2006

The dispersal and transport of pollen from numerous taxa are influenced by several meteorological factors, such as wind, rainfall, and especially temperature (Solomon and Mathews 1978; Gioulekas et al. 2004). On a seasonal timescale, temperature affects pollen production. Total airborne pollen grains in May and June reached 92.72 % (average of 5 years) of the whole year’s sum in Tianchi. Pearson correlation coefficients and significance levels between monthly pollen counts of May and June and meteorological data (monthly average temperature and monthly precipitation) in Tianchi can be seen in Table 4, revealing a very significant relationship between temperatures and pollen account, coinciding with the observations made by Wu (1983). They found that the most important factor that influences and controls the distribution and growth of spruce forests is summer temperature. Especially, 11.2 °C is suggested to be the lowest average May and June temperatures for the spruce forests to grow and the maximum error is ± 1.1 °C. Correlation with rainfall is negative because rain can wash pollen out of the air, leading to lower pollen dispersion, higher pollen accounts usually occurred with less rain. This explains why the pollen data for 2002–2003 (Figs. 3, 4; Table 2) were lower than for other years. The average temperature was the lowest but the rainfall was the highest (Fig. 5) in all of these years. The low temperature affects the production of *Picea* pollen and the high precipitation affects pollen dispersal.

**Table 4:** Pearson correlation coefficients and significance levels for the monthly pollen count and average temperature and precipitation of May and June during the 5-year period of study

Pollen	Temperature	Precipitation
Tianchi	.934*	−.379
Fukang	.821	−.291
Beishawo	.983**	−.481

* Correlation is significant at the 0.05 level

** Correlation is significant at the 0.01 level

A number of studies have made compelling arguments that plant phenology is shifting in response to global environmental change (Cleland et al. 2007). These shifts in timing of plant activity provide valuable confirmation that species as well as ecosystems are being affected by global change. However, there is little research on how plants respond to climate and climate change on high mountains in extremely arid regions.

In the past decades, a warming trend occurred in Xinjiang. During the last 50 years, an increase in air temperature with a linear tendency of 0.2 °C/10a has been observed in this region (Dai et al. 2007). Studies using regional climate models have shown that this warming trend will continue in the future, and compared with that in the 1990 s, the mean temperature in 2050 will be warmer by 1.9 to 2.3 °C (Xu et al. 2008). Under current global warming trends, how do desert plants of different ecotypes respond to the climate change? We are unsure of the extent to which climate change may affect the agriculture, forestry, and animal husbandry in the Xinjiang region.

With climate warming, the plant phenology in Northwest China desert area has an advancing trend (Chang et al. 2010), the plants’ growing periods became longer. The spring phenology of mesophytes advanced and the autumn phenology of xerophytes were delayed; the starting dates of spring phenophase of mesophytes and xerophytes differed significantly and both showed an advancing trend in the desert area. The increase of the accumulated temperature resulted in the increase of the duration days of the plants’ growing periods. In the Xinjiang region, the inclination rate between the deciduous end and growing season in the main woody plants, for example *Populus nigra var. italica* (Lombardy poplar), *Salix* (willow), *Populus bolleana Lauche* (Xinjiang poplar), and *Ulmus* (elm) is positive, which indicated phonological phenomena of woody plants occurred earlier with the climate change (Zhang et al. 2011). They also found that the most important cash crop, cotton, was sown earlier in Xinjiang. However, until now, there was no report about how pollen season responds to climate change in China.

We see from the result that *Picea* in Xinjiang showed a trend toward a 7-day shift to an earlier start of the pollen season between 2001 and 2006. The results of the correlation analysis between the pollen count, temperature, and rainfall in Tianchi station showed that the average temperature of May and June was positively correlated with *Picea* pollen production (Table 4). Jiang et al. (2011) demonstrated a lengthening of the growing season by 2.5 days/decade in Xinjiang in the past 50 years. We study the effects of climate change on the pollen season length at Tianshan Mountains. The results showed a delay of 6 days at the end of the pollen season. Also, the pollen season has become nearly 12 days longer. This reveals that the response of the vegetation in mountain regions of arid areas is extremely sensitive to climate change. A better understanding of variations and trends of the parameters of the plant growing season in this region should be scientifically important to agricultural and husbandry management and for the protection of the natural environments in arid regions.

## Conclusions

Results of this study demonstrated that over the period 2001–2006, the *Picea* pollen dispersal occurs at regular intervals along the year and especially between May and July each year. With the highest average counts for each year during the summer and the spring, while the lowest average grains were found during the winter. The results also confirmed that airborne *Picea* pollen disperses extensively and is transported widely in desert region. The number of pollen grains decline with increasing distance from the source in the pollination period. However, in this study, airborne *Picea* pollen grains began to increase and peaked suddenly in concentration in Fukang, especially in Beishawo, in March and April. Furthermore, the annual *Picea* pollen indices did not decline even as distance increased from the *Picea* forest may be attributable to the windy events can deflate the ground surface and pick up the deposited pollen grain from the topsoil into the air streams. As such, we think that these extreme weathers have significant impacts on the pollen dispersal and deposition, complicating the normal and natural courses. More pollen traps are needed to enlarge the current pollen database in order to track the exact pathways of pollen transport and hence the influence of the sandstorms and other extreme windy weathers.

The *Picea* pollen season in Tianshan Mountains showed a marked shift toward an earlier season. The duration of the *Picea* pollen season has been increasing in recent year in high mountain regions. Statistical correlation analysis showed that the temperature of May and June was positively correlated with the *Picea* pollen production. This is consistent with the recent Intergovernmental Panel on Climate Change projections regarding enhanced warming as a function of mountain regions. If similar warming trends accompany long-term climate change, it will pose a number of potential risks to mountain ecosystems in extremely arid regions. However, long-term trends cannot be discerned with this relatively short data set. This work contributes not only providing useful information for the interpretation of fossil *Picea* pollen records but also widens the existing knowledge of arid regions.
